# The effect of cultivar, sowing date and transplant location in field on bolting of Welsh onion (*Allium fistulosum* L.)

**DOI:** 10.1186/1471-2229-13-154

**Published:** 2013-10-07

**Authors:** Yinxin Dong, Zhihui Cheng, Huanwen Meng, Hanqiang Liu, Cuinan Wu, Abdul Rehman Khan

**Affiliations:** 1 College of Horticulture, Northwest A&F University, Yangling, Shaanxi 712100, People’s Republic of China

**Keywords:** Bolting, Cultivar, Seedling characteristics, Sowing date, Temperature, Transplant location, Welsh onion

## Abstract

**Background:**

Bolting reduces the quality and commercial yield of Welsh onion (*Allium fistulosum* L.) in production. However, seed production is directly dependent on flower induction and bolting. The Welsh onion belongs to the green plant vernalisation type, specific seedling characteristics and sufficient accumulated time at low temperature are indispensible for the completion of its vernalisation process. Only if these conditions for vernalisation are fulfilled, the plants will bolt in the following year. The present investigation evaluated the effects of cultivar, sowing date and transplant location in field on the bolting of Welsh onion at the Horticultural Farm of the College of Horticulture, Northwest A&F University, Yangling, Shannxi Province, China in two succeeding production years: 2010–2011 and 2011–2012. A strip split plot layout within a randomised complete block design with three replications was used.

**Results:**

The results revealed that all three factors (cultivar, sowing date and transplant location) and their interaction had significant effects on the initiation and final rate of bolting observed by 30 April. The earliest bolting date (14 February, 2011 and 15 February, 2012) and the highest bolting rate (100% in 2011 and 62% in 2012) occurred when the JinGuan cultivar was sown on 20 August and transplanted in a plastic tunnel, whereas the latest date and lowest rate (no bolting observed until 30 April) of bolting occurred when the XiaHei cultivar was sown on 29 September and transplanted in an open field.

**Conclusions:**

These results suggest that we can control bolting in Welsh onion production by choosing an appropriate cultivar, sowing date and transplant location. Choosing a late bolting cultivar, such as cultivar XiaHei, sowing around October, and transplanting in the open field can significantly delay bolting, while a sowing date in late August should be selected for seed production, and the seedlings should be transplanted in a plastic tunnel to accelerate development of the flower buds.

## Background

The Welsh onion (*Allium fistulosum* L.) originated in Southwest China and is widely cultivated in East Asian countries, i.e., China, Japan and Korea [1]. China ranks first in the world in Welsh onion production, in 2011, the onion (including Welsh onion) has approximately 28 thousand hectares in cultivation and a total production of 979 thousand tons (FAO, 2011, see Additional file 1).

Vernalisation is a prolonged exposure to low but non-freezing temperatures that is required by many biennial plants in temperate climates to enter their reproductive phase. This process has been studied widely in several crops such as winter wheat (*Triticum aestivum* L.) [2,3], sugar beet (*Beta vulgaris* L.) [4], broccoli (*Brassica oleracea* var. *italica*) [5], onion (*Allium cepa* L.) [6-10], radish (*Raphanus sativus* L.) [11], *Brassica rapa* (syn. *campestris*) [12] and lily (*Lilium* spp.) [13,14]. The Welsh onion requires a low temperature period or vernalisation to flower. Vernalisation in the Welsh onion has been of research interest because of its significance for the control of bolting in plant breeding, seed production and crop productivity. On the one hand, farmers producing Welsh onion must prevent bolting because the bolting will lower the Welsh onion’s quality and commercial yield. On the other hand, seed producers prefer to induce flowering to shorten the period of seed production because seed set is directly dependent upon flower induction and bolting [15]. Although there are a few reports concerning vernalisation studying in Welsh onions [16,17], those research mainly pay attention on the plant growth regulators’ effect on plant bolting or before transplant, certain cultivar’s accumulated temperature needed for bolting, the factors that initiate Welsh onion flowering, such as cultivar, sowing date and transplant location, are far from fully understood, and more research is urgently needed.

In most areas of North China, the Welsh onion crop always experiences winter temperatures that are low enough to induce premature flowering or bolting. Flower induction is controlled by temperature and daylength. For Welsh onion, low temperatures and short days induce flowering [9], but the cold requirement is different with the origin of cultivars and depends, in other species, upon whether the cultivars have an obligate or facultative vernalisation requirement [11]. Flowering of Welsh onion is generally induced by temperatures below 13°C, when seedlings have formed a certain number of leaves or a stem of a certain thickness [18]. The Welsh onion belongs to the green plant vernalisation type, which is characterised by seedlings that must be grown to a certain age before they can sense the cold environment and begin the process of vernalisation. The characteristics of transplants also determine the establishment rate of plants and their ability to respond to low temperature [19]. Moreover, the response of plants to vernalisation is determined not only by the temperature during the vernalisation period but also by the duration of the low-temperature exposure [20,21], and thus, the cold requirements for vernalisation vary greatly with the genotype and the environment [22]. Therefore, specific seedling characteristics and sufficient accumulated time at low temperatures are needed by the Welsh onion to complete the vernalisation process. If these conditions for vernalisation are fulfilled, then the plants bolt the following year.

The sowing-date recommendations for fall-seeded cultivars are based on the resistance of the cultivar to bolting [23]. Bolting-susceptible cultivars are seeded in late September, so that the plants are smaller going into the winter season and thus less susceptible to vernalisation temperatures and less likely to bolt than earlier-seeded, larger plants; however, these smaller plants produce lower yields. As the cultivars are seeded earlier in the fall, the larger plants produced before winter have a higher yield potential the next growing season but are also more likely to bolt, as researchers have observed that fall-sown onions increase their bolting rate with earlier seeding dates [24]. The proper date for fall sowing that minimises bolting and maximises yield can be difficult to determine because the date is dependent on both the cultivar and the environment. Cultivars resistant to bolting can be sown earlier. Farmers producing Welsh onions can also choose different transplant locations for the winter. To advance maturity and market the crop earlier, farmers often sow early and sometimes choose a plastic tunnel as the transplant location; however, attempts to meet an earlier marketing date may simultaneously produce unwanted, premature and heavy bolting. It is clear that variation in production practices that involve the cultivar, sowing date and transplant location significantly affect the tendency of Welsh onion to bolt, but the specific effect of each factor and their interactions have not been investigated. To identify the effects of these variables on bolting, ten major cultivars, including types that grow year-round in North China, were selected and evaluated for differences in sensitivity to vernalisation. By sowing on three dates, the seedlings with different characteristics were transplanted simultaneously to three locations that provided different low-temperature accumulations.

The objective of this study is to clarify how bolting of the Welsh onion is affected by the factors of (a) cultivar (genotype), (b) sowing date (seedling characteristics), (c) transplant location (accumulated low temperature units) in field and their interactions. This investigation can facilitate an understanding of the bolting characteristics of China's main Welsh onion cultivars and how these factors control inflorescence initiation and development. Simultaneously, the data generated can be used, on the one hand, to devise techniques for preventing flowering in Welsh onion production and, on the other hand, to help seed producers comprehend and promote Welsh onion flowering and, therefore, accelerate seed production.

## Results

### Effect of sowing date and accumulated temperature on seedling characteristics

Because the plants were sown on three different dates, there were 50-, 70- and 90-d-old seedlings on the day of transplantation. The seedlings with different sowing dates also experienced a different active accumulated temperature (AAT) and effective accumulated temperature (EAT) (Table 1) before transplantation, transplanted on 20 August was highest in both AAT and EAT, followed by transplanted on 9 September and 29 September. Seedling-age and accumulated-temperature have positive effect on seedling morphology with limits. The seedling morphology of the test cultivars, especially the pseudostem diameter, varied significantly among the sowing dates in both years (Table 2) for the seedling-age and accumulated-temperature reason.

**Table 1:** Seedling age, active accumulated temperature (AAT) and effective accumulated temperature (EAT) of ≥5° C and ≥10°C from the sowing date to 19 November, 2010 and 2011

**Sowing date (mm. dd)**	**Seedling age (d)**	**AAT (°C)**		**EAT (°C)**	
		**≥5°C**	**≥10°C**	**≥5°C**	**≥10°C**
2010					
08.20(D_1_)	90	1575.0	1470.3	1100.0	660.3
09.09(D_2_)	70	1156.6	1051.9	796.6	441.9
09.29(D_3_)	50	738.2	633.5	478.2	223.5
2011					
08.20(D_1_)	90	1481.1	1413.7	1026.1	563.7
09.09(D_2_)	70	1055.5	997.7	690.5	347.7
09.29(D_3_)	50	719.5	652.1	464.5	202.1

mm: month.

dd: date.

**Table 2:** Plant height, pseudostem diameter and leaf number on 19 November, 2010 and 2011, for the tested cultivars sown on three different dates

**Cultivar**	**Plant height(cm)**			**Pseudostem diameter(mm)**			**Leaf number**		
	**D**_**1**_	**D**_**2**_	**D**_**3**_	**D**_**1**_	**D**_**2**_	**D**_**3**_	**D**_**1**_	**D**_**2**_	**D**_**3**_
2010									
C_1_	16.4 ± 0.5a	12.3 ± 0.5b	11.9 ± 0.6b	5.91 ± 0.36a	4.64 ± 0.17b	2.31 ± 0.07c	3.7 ± 0.2a	3.6 ± 0.2a	2.9 ± 0.1b
C_2_	25.7 ± 0.8a	12.7 ± 0.5b	12.0 ± 0.6b	6.96 ± 0.55a	4.82 ± 0.22b	1.75 ± 0.12c	4.0 ± 0.0a	3.2 ± 0.1b	2.6 ± 0.2c
C_3_	25.8 ± 0.6a	15.9 ± 0.7b	13.0 ± 0.3c	5.13 ± 0.21a	4.06 ± 0.15b	1.74 ± 0.10c	4.0 ± 0.0a	3.5 ± 0.2b	2.3 ± 0.2c
C_4_	24.2 ± 1.1a	15.1 ± 0.5b	11.9 ± 0.4c	6.50 ± 0.17a	5.58 ± 0.11b	2.72 ± 0.08c	3.5 ± 0.2a	3.3 ± 0.2ab	3.0 ± 0.0b
C_5_	18.3 ± 0.8a	15.3 ± 1.2b	12.0 ± 0.3c	7.15 ± 0.30a	6.02 ± 0.17b	2.49 ± 0.55c	3.2 ± 0.1a	3.0 ± 0.0a	2.6 ± 0.3b
C_6_	21.2 ± 1.7a	14.0 ± 0.6b	12.5 ± 0.7b	5.90 ± 0.34a	4.50 ± 0.19b	2.35 ± 0.10c	3.4 ± 0.2a	3.4 ± 0.2a	2.9 ± 0.1a
C_7_	22.7 ± 0.7a	14.6 ± 0.5b	12.2 ± 0.7c	7.07 ± 0.59a	4.71 ± 0.34b	2.49 ± 0.14 c	4.0 ± 0.0a	3.4 ± 0.2b	3.2 ± 0.2b
C_8_	19.7 ± 0.6a	14.9 ± 0.8ab	13.1 ± 0.5b	5.66 ± 0.44a	4.70 ± 0.64b	2.95 ± 0.18c	3.5 ± 0.2a	3.0 ± 0.0b	2.3 ± 0.2c
C_9_	24.0 ± 1.1a	15.2 ± 0.5b	11.1 ± 0.4c	6.47 ± 0.32a	5.28 ± 0.28b	2.76 ± 0.13c	3.6 ± 0.2a	3.5 ± 0.2a	2.9 ± 0.1b
C_10_	25.1 ± 1.1a	12.4 ± 0.6b	10.6 ± 0.6b	7.28 ± 0.46a	5010 ± 0.20b	2.54 ± 0.13c	3.2 ± 0.1a	3.1 ± 0.1a	3.0 ± 0.0a
2011									
C_1_	20.6 ± 1.2a	13.2 ± 0.8b	9.6 ± 0.5c	4.37 ± 0.25a	2.12 ± 0.07b	1.55 ± 0.99c	3.2 ± 0.1a	2.7 ± 0.2b	2.6 ± 0.2b
C_3_	23.0 ± 1.2a	14.0 ± 0.7b	10.0 ± 0.4c	3.57 ± 0.15a	2.21 ± 0.13b	1.69 ± 0.08c	3.0 ± 0.0a	3.0 ± 0.1a	2.7 ± 0.2b
C_6_	18.1 ± 1.0a	11.8 ± 1.0b	9.8 ± 0.4c	3.65 ± 0.23a	2.17 ± 0.08b	1.57 ± 0.08c	3.3 ± 0.1a	3.1 ± 0.1ab	3.0 ± 0.1b
C_7_	20.1 ± 1.0a	12.6 ± 0.8b	8.9 ± 0.4c	3.90 ± 0.20a	2.53 ± 0.13b	1.32 ± 0.12c	3.4 ± 0.2a	3.1 ± 0.1b	2.4 ± 0.2c
C_8_	19.5 ± 0.9a	13.1 ± 0.5b	10.3 ± 0.5c	3.51 ± 0.24a	2.35 ± 0.11b	1.83 ± 0.08c	3.4 ± 0.2a	3.4 ± 0.2a	2.7 ± 0.2b
C_10_	19.4 ± 1.2a	12.5 ± 0.5b	7.5 ± 0.4c	4.08 ± 0.39a	2.45 ± 0.06b	1.30 ± 0.05c	3.4 ± 0.2a	3.3 ± 0.2a	2.1 ± 0.1b

Values are means ± standard errors. Different letters within rows and plant parameters indicate significant differences between means of different sowing dates at the 5% probability level by LSD.

### Different temperatures in three transplant locations from 19 November to 30 April in the 2 successive production years

From the hourly temperature records, the daily average temperature was obtained and the active accumulated temperature (AAT), effective accumulated temperature (EAT) of ≥5°C, ≥10°C and ≥15°C, accumulated low temperature (ALT) and effective accumulated low temperature (EALT) of ≤13°C in different transplant locations were calculated from 19 November to 30 April for each year of the experiment. There was a significant difference in the four temperature indictors among the three different transplant locations (Table 3). The highest AATs and EATs of ≥5°C, ≥10°C and ≥15°C were in the plastic tunnel (In the first year, AAT of ≥5°C, ≥10°C and ≥15°C were 1885.8°C, 1539.6°C, and 1027.7°C, respectively; EAT of ≥5°C, ≥10°C and ≥15°C were 122.08°C, 659.6°C and 337.3°C, respectively. In the second year, AAT of ≥5°C, ≥10°C and ≥15°C were 1047.3°C, 1060.1°C, and 924.9°C, respectively; EAT of ≥5°C, ≥10°C and ≥15°C were 920.3°C, 490.1°C and 219.9°C, respectively) and the lowest were in the open field (In the first year, AAT of ≥5°C, ≥10°C and ≥15°C were 912.6°C, 611.5°C, and 390.5°C; EAT of ≥5°C, ≥10°C and ≥15°C were 487.6°C, 221.5°C and 75.5°C, respectively. In the second year, AAT of ≥5°C, ≥10°C and ≥15°C were 758.8°C 603.0°C, and 420.5°C, respectively; EAT of ≥5°C, ≥10°C and ≥15°C were 473.8°C, 233.0°C and 63.5°C, respectively) in both years. However, noticeable patterns among the ALT and EALT values of the three locations in the two year were: the lowest ALT (≤13°C) and the highest EALT (≤13°C) were in the open field (200.7°C and 1554.3°C in the first year and 445.3°C and 1384.7°C in the second year) and the highest ATL (≤13°C) and the lowest EALT (≤13°C) were in the plastic tunnel (801.3°C and 589.7°C in the first year and 618.0°C and 812.0°C in the second year).

**Table 3:** Active accumulated temperature (AAT), effective accumulated temperature (EAT) of 5°C, 10°C and 15°C, accumulated low temperature (ALT) and effective accumulated low temperature (EALT) at 13°C in different transplant locations from 19 November to 30 April, in the two succeeding production years

**Location**	**AAT(°C)**			**EAT(°C)**			**ALT(°C)**	**EALT(°C)**
	**≥5°C**	**≥10°C**	**≥15°C**	**≥5°C**	**≥10°C**	**≥15°C**	**≤13°C**	**≤13°C**
2010-2011								
L_1_	912.6	611.5	390.5	487.6	221.5	75.5	200.7	1554.3
L_2_	1367.8	940.9	704.5	812.8	400.9	179.5	501.2	1058.8
L_3_	1855.8	1539.6	1027.7	1220.8	659.6	337.7	801.3	589.7
2011-2012								
L_1_	758.8	603.0	420.5	473.8	223.0	63.5	445.3	1348.7
L_2_	1134.5	867.4	681.9	689.5	357.4	126.9	584.4	1053.6
L_3_	1470.3	1060.1	924.9	920.3	490.1	219.9	618.0	812.0

### Comparison of the bolting characteristics of the ten cultivars

The number of days from the transplantation date to the initiation of bolting differed among the cultivars (Table 4). In both of the two years, with the three transplanted locations and the three sowing dates (except 29 September, 2011 sown and transplanted in open field) treatments, the bolting dates of C_1_ and C_7_ were significantly earlier and those of C_3_ and C_10_ were significantly later than the bolting dates of the other cultivars. There was no significant difference among the remaining six cultivars, and thus, it was inferred that C_3_ and C_10_ were highly resistant to bolting and were categorised as a strong bolting type, C_6_ and C_8_ were moderately susceptible to bolting and were classified as a moderate bolting type, C_1_ and C_7_ were highly susceptible to bolting and termed a weak bolting type. The same classification was used during the next year of the experiment, i.e., 2011–2012. To simplify the analysis, the C_1_, C_3_ and C_6_ cultivars were selected as representative of each of the above bolting types.

**Table 4:** Days from transplant to initial bolting for ten cultivars, three sowing dates and three locations in 2011

**Cultivar**	**L**_**1**_**(d)**			**L**_**2**_**(d)**			**L**_**3**_**(d)**		
	**D**_**1**_	**D**_**2**_	**D**_**3**_	**D**_**1**_	**D**_**2**_	**D**_**3**_	**D**_**1**_	**D**_**2**_	**D3**
C_1_	137.0 ± 1.0de	143.0 ± 2.0 cd	-	86.0 ± 1.0d	98.0 ± 2.0c	118.0 ± 2.0d	77.0 ± 1.0d	85.0 ± 1.0c	89.0 ± 1.0d
C_2_	141.0 ± 1.7 cd	147.0 ± 0.0b	-	104.0 ± 2.0b	118.0 ± 1.0b	133.0 ± 4.0c	102.0 ± 1.7bc	104.0 ± 2.0b	122.0 ± 1.0c
C_3_	-a	-a	-	131.0 ± 3.6a	146.0 ± 2.0a	-a	107.0 ± 5.0ab	127.0 ± 1.0a	134.0 ± 5.3b
C_4_	141.0 ± 1.7 cd	147.0 ± 0.0b	-	108.0 ± 3.5b	115.0 ± 2.6b	137.0 ± 2.0c	102.0 ± 1.7bc	106.0 ± 2.0b	115.0 ± 6.6c
C_5_	143.0 ± 1.0c	147.0 ± 1.7b	-	110.0 ± 4.0c	115.0 ± 1.0b	142.0 ± 2.0bc	98.0 ± 1.0	107.0 ± 5.0b	126.0 ± 6.0bc
C_6_	143.0 ± 1.0c	147.0 ± 0.0b	-	114.0 ± 0.0b	115.0 ± 1.0b	140.0 ± 3.6bc	102.0 ± 3.0	104.0 ± 1.0b	123.0 ± 0.0bc
C_7_	135.0 ± 0.0e	140.0 ± 1.0d	-	85.0 ± 4.0d	97.0 ± 2.6c	121.0 ± 1.0d	84.0 ± 3.0c	89.0 ± 1.0c	93.0 ± 3.0d
C_8_	141.0 ± 1.7cd	145.0 ± 1.0bc	-	106.0 ± 4.0bc	116.0 ± 2.0b	137.0 ± 2.0c	99.0 ± 0.0bc	102.0 ± 3.5b	118.0 ± 2.6c
C_9_	144.0 ± 1.7c	147.0 ± 1.7b	-	114.0 ± 0.0b	119.0 ± 2.6b	134.0 ± 3.6c	102.0 ± 1.7d	110.0 ± 2.0b	118.0 ± 1.0c
C_10_	148.0 ± 1.0b	-a	-	120.0 ± 1.7a	129.0 ± 1.7a	140.0 ± 2.6a	113.0 ± 2.6a	131.0 ± 2.6a	142.0 ± 1.0a

Values are means ± standard errors. Different letters within columns indicate significant differences between means of different cultivars at the 5% probability level by LSD.

- a stands for no bolting, where 3 May, 2011, (156 days) was used as the bolting date for the LSD tests.

### The influence of cultivar, sowing date and transplant location on Welsh onion bolting

The number of days from the transplantation to the initial bolting, the number of days to the 50% bolting rate and the bolting rate by 30 April were selected as indicators to evaluate the bolting behaviour. To simplify the analysis, the C_1_, C_3_ and C_6_ cultivars were selected as representative of different bolting types as described above. The results of each treatment are listed in Table 5 for 2011 and 2012. The effects of each factor and their interactions were analysed.

**Table 5:** Days from transplant to initial bolting, to 50% bolting and bolting rate by 30 April for each treatment and year

**Cultivar**	**Sowing date**	**Transplant location**	**Days from transplant to initial bolting (d)**		**Days from transplant to 50% bolting (d)**		**Bolting rate by 30 April (%)**	
			**2011**	**2012**	**2011**	**2012**	**2011**	**2012**
C_1_	T_1_	L_1_	137.0 ± 1.0	117.0 ± 1.0	-	-	41.0 ± 3.2	29.0 ± 2.7
		L_2_	86.0 ± 1.0	87.0 ± 3.6	119.0 ± 1.0	-	100.0 ± 0.0	56.0 ± 2.5
		L_3_	77.0 ± 1.0	78.0 ± 1.0	113.0 ± 5.6	-	100.0 ± 0.0	62.3 ± 2.6
	T_2_	L_1_	143.0 ± 2.0	143.0 ± 1.0	-	-	14.7 ± 2.2	6.3 ± 1.5
		L_2_	98.0 ± 2.0	120.0 ± 3.6	143.0 ± 1.0	-	59.3 ± 4.1	18.3 ± 0.9
		L_3_	85.0 ± 1.0	114.0 ± 4.0	128.0 ± 2.0	-	85.0 ± 4.5	28.3 ± 2.0
	T_3_	L_1_	-	154.0 ± 0.0	-	-	0	1.3 ± 0.3
		L_2_	117.0 ± 2.0	130.0 ± 6.9	-	-	15.3 ± 1.3	14.3 ± 2.4
		L_3_	89.0 ± 1.0	124.0 ± 1.7	122.0 ± 2.6	-	69.7 ± 8.2	24.3 ± 1.8
C_3_	T_1_	L_1_	-	-	-	-	0	0
		L_2_	131.0 ± 3.6	151.0 ± 1.7	150.0 ± 3.5	-	58.3 ± 5.4	7.0 ± 2.7
		L_3_	107.0 ± 5.0	143.0 ± 1.0	139.0 ± 3.6	-	87.7 ± 2.7	23.0 ± 1.0
	T_2_	L_1_	-	-	-	-	0	0
		L_2_	146.0 ± 2.0	155.0 ± 1.0	-	-	43.0 ± 6.0	5.0 ± 0.6
		L_3_	127.0 ± 1.0	151.0 ± 1.7	149.0 ± 2.6	-	66. 7 ± 8.8	18.0 ± 2.3
	T_3_	L_1_	-	-	-	-	0	0
		L_2_	-	-	-	-	0	0
		L_3_	134.0 ± 5.3	-	-	-	8.0 ± 3.1	0
C_6_	T_1_	L_1_	143. ± 1.0	133.0 ± 3.5	-	-	20.7 ± 4.8	9.0 ± 4.7
		L_2_	114.0 ± 0.0	131.0 ± 1.0	125.0 ± 2.6	-	87.7 ± 6.4	29.3 ± 3.5
		L_3_	102.0 ± 3.0	112.0 ± 3.5	122.0 ± 2.6	-	85.0 ± 5.5	55.3 ± 1.9
	T_2_	L_1_	147.0 ± 0.0	156.0 ± 1.0	-	-	7.7 ± 2.2	5.0 ± 1.5
		L_2_	115.0 ± 1.0	151.0 ± 1.7	144.0 ± 1.7	-	36.7 ± 11.3	12.7 ± 4.6
		L_3_	104.0 ± 1.0	121.0 ± 4.6	132.0 ± 1.7	-	44.7 ± 8.7	19.3 ± 1.5
	T_3_	L_1_	-	-	-	-	0	0
		L_2_	140.0 ± 3.6	155.0 ± 1.0	-	-	2.0 ± 0.0	1.3 ± 0.3
		L_3_	123.0 ± 0.0	149.0 ± 1.0	-	-	40.0 ± 6.5	7.3 ± 1.2

Values are means ± standard errors.

- Did not bolt.

### Main effects of cultivar, sowing date and transplant location on the indicators of bolting

All the factors, including the cultivar, sowing date and transplant location, had a significant influence in both years on the days from transplant to initial bolting, the days from transplant to 50% bolting and the bolting rate by the 30 April.

The interactions of cultivar * sowing date (C*D) and sowing date * transplant location (D*L) had a significant influence on the days from transplant to initial bolting (2011, 2012), the days from transplant to 50% bolting (2011) and the bolting rate by 30 April (2011, 2012). Because some of the treatments did not attain 50% bolting in 2012, there was no significant difference in 2012 among the treatments in the days from transplant to 50% bolting. The interaction of cultivar * transplant location (C*L) on the days from transplant to 50% bolting were not significant in either of the two years.

The interaction of variety * sowing date * transplant location (C*D*L) on the days from transplant to 50% bolting was not significant in either year, but there was a significant influence on the days from transplant to initial bolting and the bolting rate by 30 April in both 2011 and 2012 (Table 6).

**Table 6:** The effects of cultivar, sowing date, and transplant location on days from transplant to initial bolting, to 50% bolting and bolting rate by 30 April in 2011 and 2012

**Treatment**	**Days from transplant to initial bolting (d)**		**Days from transplant to 50% bolting (d)**		**Bolting rate by 30 April (%)**	
	**2011**	**2012**	**2011**	**2012**	**2011**	**2012**
Grand mean	128.4	139.7	140.5	-	39.7	16.0
Cultivars						
JinGuan	110.9c^a^	118.6c	125.8c	-	53.9a	26.7a
XiaHei	145.2a	158.9a	-a	-	29.3c	5.9c
YeFu	129.1b	141.6b	130.8b	-	36.0b	15.5b
Sowing date						
08.20	118.0c	124.2c	119.8c	-	64.5a	30.1a
09.09	125.6b	141.9b	136.8b	-	39.7b	12.6b
09.29	141.7a	152.9a	-a	-	15.0c	5.4c
Transplant location						
Open field	155.0a	151.9a	-a	-	9.3c	5.6c
Cold shed	123.7b	138.4b	132.8b	-	44.7b	16.0b
Plastic tunnel	106.6c	128.7c	123.8c	-	65.2a	26.4a
F-test						
Cultivar (C)	***	***	***	NS	***	***
Sowing date (D)	***	***	***	NS	***	***
Transplant location (L)	***	***	***	NS	**	***
C*D	***	***	*	NS	***	***
C*L	***	***	NS	NS	*	***
D*L	***	***	**	NS	***	***
C*D*L	***	***	NS	NS	***	***

* Significant at P = 0.05.

** Significant at P = 0.01.

*** Significant at P = 0.001.

NS, Not significant.

^a^Different letters indicate significant differences between means within columns at the 5% probability level by LSD.

-a stands for no bolting, where 3 May (165 days for 2011 and 166 d for 2012) was used as the bolting date for LSD tests.

Among the three analysed cultivars in 2011, the JinGuan (C_1_) cultivar was the first to bolt and, on average, did so by 10 May, only 111 days after sowing, which was 18 days earlier than the YeFu (C_6_) cultivar and 34 days earlier than the XiaHei (C_3_) cultivar. Moreover, JinGuan (C_1_) attained 50% bolting by 126 days after transplant; YeFu (C_6_) required 131 days, and XiaHei (C_3_) did not reach 50% bolting until 30 April. As for the bolting rate, JinGuan (C_1_), YeFu (C_6_) and XiaHei (C_3_) attained levels of 54, 36 and 29%, respectively, by 30 April.

In 2011, the plants sown on 20 August (D_1_) bolted by approximately 17 March. This date was 8 days earlier than that of the plants sown on 9 September (D_2_) and 24 days earlier than that of the plants sown on 29 September (D_3_). A period of 120 days was required from transplant to 50% bolting when the plants were sown on 20 August (D_1_), which was 27 days fewer than was required by the plants sown on 9 September (D_2_). The plants sown on 29 September (D_3_) did not reach 50% bolting until 30 April. The bolting rates were 65, 40 and 15%, respectively, for the earliest, middle and latest of the three sowing dates.

The use of the open field (L_1_) as the Welsh onion transplant location delayed bolting by approximately 49 and 21 days, respectively, compared with the use of the plastic tunnel (L_3_) and the cold shed (L_2_). The open field (L_1_) treatment produced bolting on approximately 23 April. The time required from transplantation to 50% bolting was 124 days in the plastic tunnel (L_3_) and 133 days in the cold shed (L_2_). In the open field, the Welsh onions did not reach 50% bolting until 30 April. The final bolting rate by 30 April in the open field (L_1_), cold shed (L_2_) and plastic tunnel (L_3_) was 9, 45, and 65%, respectively.

In 2012 compared with 2011, the dates when the bolting started and reached the 50% level were slightly later, and the bolting rate on 30 April was lower. However, the overall effects on bolting of the different cultivars, sowing dates, transplant locations and factor interactions were similar in the two years.

Thus, except the interaction of cultivar * transplant location (C*L) and the interaction of variety * sowing date * transplant location (C*D*L) on the days from transplant to 50% bolting were not significant in either of the two years, other factors, including the cultivar, sowing date and transplant location and their interaction, had a significant influence in both years on the days from transplant to initial bolting, the days from transplant to 50% bolting and the bolting rate by the 30 April.

### Effects of cultivar and sowing date on days from transplant to initial bolting, days to 50% bolting and bolting rate by 30 April in the two years

The interaction between the cultivar and the sowing date significantly influenced the initial bolting date (2011, 2012) and the bolting rate by 30 April (2011). In 2011, the earliest initial and 50% bolting rate occurred when the JinGuan cultivar was sown on 20 August (C_1_*D_1_). It only took 100 (27 February) and 116 days (15 May) for the initial and 50% bolting levels to occur, respectively, and the bolting rate reached 80%, which was the highest in all of the treatments. By contrast, the treatment showing the latest bolting was the XiaHei cultivar, which was sown on 29 September (C_3_*D_3_). This cultivar required 155 days (23 April) for the initial bolting and did not reach the 50% bolting level until 30 April, on average. The final bolting rate of this cultivar was lower than 3%, which was the lowest among all of the treatments (Table 7).

**Table 7:** Effects of cultivar and sowing date on days from transplant to initial bolting, to 50% bolting and bolting rate by 30 April in 2011 and 2012

**Treatment**	**Days from transplant to initial bolting (d)**		**Days from transplant to 50% bolting (d)**		**Bolting rate to 30 April (%)**	
	**2011**	**2012**	**2011**	**2012**	**2011**	**2012**
Grand mean	128.4	139.7	148.7	-	39.7	16.2
C_1_*D_1_	100.0	94.0	116.0	-	80.3	49.1
C_1_*D_2_	108.7	125.7	135.5	-	53.0	17.7
C_1_*D_3_	124.0	136.0	-	-	28.3	13.3
F-test	***	***	***	NS	***	***
C_3_*D_1_	134.3	153.3	-	-	48.7	10.00
C_3_*D_2_	146.0	157.3	-	-	36.6	7.7
C_3_*D_3_	155.3	-	-	-	2.7	0
F-test	***	***	NS	NS	***	***
C_6_*D_1_	119.7	125.3	123.5	-	64.4	31.2
C_6_*D_2_	122.0	142.7	138.0	-	29.7	12.3
C_6_*D_3_	145.7	156.7	-	-	14.0	2.9
F-test	***	***	***	NS	***	***

*** Significant at P = 0.001.

NS, Not significant.

-a stands for no bolting, where 3 May (165 days for 2011 and 166 d for 2012) was used as the bolting date for LSD tests.

The days to initial bolting were similar in 2011 and 2012. In 2012, however, the 50% bolting date did not differ significantly among the treatments because none of the treatments reached the 50% bolting rate by 30 April. Surprisingly, the XiaHei (C_3_) cultivar did not reach 50% bolting in any of the three sowing dates in either 2011 or 2012.

### Effects of cultivar and transplant location on days from transplant to initial bolting, days to 50% bolting and bolting rate by 30 April in two years

There was a significant difference among the treatments in the days from the transplant to the initial bolting (2011, 2012), and in the bolting rate by 30 April (2011, 2012) because of the interaction of the cultivar and the transplant location (Table 8). In particular, the JinGuan cultivar transplanted in the plastic tunnel location (C_1_*L_3_) took only 84 days in 2011 and 106 days in 2012 to initial bolting, which were the shortest times of any of the treatments. The JinGuan (C_1_) bolting rates were also the highest, i.e., 85% and 29% in 2011 and 2012, respectively. The treatment that took the longest to initiate bolting was the XiaHei cultivar that was transplanted in the open-field location (C_3_*L_1_); no bolting was observed before 30 April. Because no treatment reached 50% bolting in 2012, it was difficult to show an interaction of the cultivar and the transplant location, a situation similar to that encountered when exploring an interaction between the cultivar and the sowing date. The XiaHei (C_3_) cultivar did not reach 50% bolting in any of the three transplant locations in 2011.

**Table 8:** Effects of cultivar and transplant location on days from transplant to initial bolting, to 50% bolting and bolting rate by 30 April in 2011 and 2012

**Treatment**	**Days from transplant to initial bolting (d)**		**Days from transplant to 50% bolting (d)**		**Bolting rate to 30 April (%)**	
	**2011**	**2012**	**2011**	**2012**	**2011**	**2012**
Grand mean	128.4	139.7	148.7	-	39.7	14.9
C_1_*L_1_	148.3	138.0	-	-	18.6	12.2
C_1_*L_2_	100.7	112.3	131.0	-	58.2	28.3
C_1_*L_3_	83.7	105.3	120.5	-	84.9	29.6
F-test	***	***	***	NS	***	***
C_3_*L_1_	-	-	-	-	0	0
C_3_*L_2_	147.3	157.3	-	-	33.8	4.0
C_3_*L_3_	123.3	153.3	-	-	54.1	13.7
F-test	***	***	NS	NS	***	***
C_6_*L_1_	151.7	151.7	-		9.4	4.7
C_6_*L_2_	123.0	145.7	134.5	-	42.1	14.4
C_6_*L_3_	112.7	127.3	127.0	-	56.6	27.3
F-test	***	***	***	NS	***	***

***Significant at P = 0.001.

NS, Not significant.

-a stands for no bolting, where 3 May (165 days for 2011 and 166 d for 2012) was used as the bolting date for LSD tests.

Effects of sowing date and transplant location on days from transplant to initial bolting, days to 50% bolting and the bolting rate by 30 April in the two years.

An interaction between the sowing date and the transplant location significantly affected the bolting parameters of the Welsh onion cultivars (Table 9). In 2011, the days to initial bolting increased from 95 with the sowing on 20 August and the transplant location of the plastic tunnel (D_1_*L_3_) to above 165 with the sowing on 29 September and the open-field transplant location (D_3_*L_1_). A similar pattern appeared with the same two treatments in 2012, i.e., the days needed for initial bolting increased from 111 to 162. The D_1_*L_3_ treatment combination required the fewest days to attain the 50% bolting rate (2011) and exhibited the highest final bolting rate, whereas the D_3_*L_1_ treatment combination never reached 50% bolting (2011, 2012) and showed the lowest final bolting rate. The final bolting rates for the D_1_*L_3_ and D_3_*L_1_ treatment combinations, respectively, were 91 and 0% in 2011 and 47 and 0.44% in 2012.

**Table 9:** Effects of sowing date and transplant location on days from transplant to initial bolting, to 50% bolting and bolting rate by 30 April in 2011 and 2012

**Treatment**	**Days from transplant to initial bolting (d)**		**Days from transplant to 50% bolting (d)**		**Bolting rate to 30 April (%)**	
	**2011**	**2012**	**2011**	**2012**	**2011**	**2012**
Grand mean	128.4	139.7	130.3	-	39.7	16.0
D_1_*L_1_	148.3	138.7	-	-	20.6	12.7
D_1_*L_2_	110.3	123.0	122.0	-	82.0	30.8
D_1_*L_3_	95.3	111.0	117.5	-	90.9	46.9
F-test	***	***	***	NS	***	***
D_2_*L_1_	151.7	155.0	-	-	7.4	3.8
D_2_*L_2_	119.7	142.0	143.5	-	46.3	12.0
D_2_*L_3_	105.3	128.7	130.0	-	65.4	21.9
F-test	***	***	***	NS	***	***
D_3_*L_1_	-	162.0	-	-	0	0.4
D_3_*L_2_	141.0	150.3	-	-	5.8	5.2
D_3_*L_3_	119.0	146.3	-	-	39.2	10.6
F-test	***	**	NS	NS	***	***

** Significant at P = 0.01.

*** Significant at P = 0.001.

NS, Not significant.

-a stands for no bolting, where 3 May (165 days for 2011 and 166 d for 2012) was used as the bolting date for LSD tests.

On 29 September (D_3_), the latest sowing date, no treatment reached the 50% bolting rate in any of the three transplant locations in either 2011 or 2012, so there was no difference among these treatments.

### The influence of seedling age, AAT and EAT on bolting of the cultivar of XiaHei

Cultivar XiaHei sown on 29 September, transplanted on cold shed could not bolt in the first year compared with sown on 9 September, we can deduce that pseudostem diameter around 4.06 mm, AAT (≥5°C) of 1156.1°C and EAT (≥5°C) of 796.6°C before transplant were necessary for cultivar 3 to response the cold environment and begin the process of vernalisation, then bolting in the next year.

Taken together, sowing date affected the seedling morphology when transplanting, which would further determine Welsh onion’s bolting behaviour together with the duration of low temperature. Specifically, all three factors (cultivar, sowing date and transplant location) and their interaction had significant effects on the initial and final rate of bolting observed on 30 April. The earliest bolting date and highest bolting rate occurred when the JinGuan cultivar was sown on 20 August and transplanted in a plastic tunnel, whereas the latest date and lowest rate of bolting occurred when the XiaHei cultivar was sown on 29 September and transplanted in an open field.

## Discussion

The results from the two experimental years showed that the cultivar, sowing date and transplant location all significantly influenced the bolting of the Welsh onion. The JinGuan (C_1_) and TieGanWuTong (C_7_) cultivars could be considered early-bolting varieties, whereas the XiaHei (C_3_) and LinBang (C_10_) cultivars could be considered late-bolting varieties. Transplant characteristics are closely related to the bolting characteristics of plants [19]. In our experiment, because the seedling age and the environment conditions, such as AAT and EAT, during nursing were different for the different sowing date treatments, the morphological development of the seedlings was not the same on the transplant date, and thus, the sowing date has an influence on the bolting of the Welsh onion. The cold requirement for bolting varies greatly in relation to the genotype and the environment. For certain Welsh onion cultivars, although they were grown in low-temperature environments in the three transplant locations, the flowers were initiated or induced in a short period. Other plants behave similarly, for example, approximately two weeks at 12°C were required for flowering in Cape Daisy [25]. Once the cold requirement was fulfilled, the growth of the Welsh onion was better at high (plastic tunnel) than at low temperature (open field), as long as the high temperature was below the devernalisation temperature. Before they bolt, plants need to grow a certain number of leaves, and the Welsh onions formed a sufficient number of leaves to bolt earlier in the plastic tunnel (L_3_) than in the open field (L_1_), i.e., the Welsh onions in the plastic tunnel bolted earlier than those in the open field because they experienced a more suitable environment and grew faster. The temperature and certain seedling characteristic requirements could explain why all 3 cultivars sown on the 29 September (D_3_) and transplanted to the open field (L_1_) failed to bolt in 2011. In 2012, although the JinGuan (C_1_) cultivar bolted at a very low rate of 1.33%, this bolting could be considered a chance occurrence.

In 2012, both the initial bolting date and the date of 50% bolting were later than those for 2011, and the bolting rate by 30 April was lower than that in 2011. This pattern occurred because the temperature was lower and the rainfall higher during the nursery period in 2012 than in 2011, and thus, the transplants were smaller in 2012 than in 2011. In addition, the winter temperature in 2012 was lower than in 2011. Thus, combining these factors, there was a difference between the 2 years.

For cultivar XiaHei, through the first year’s experiment, we could deduce that pseudostem diameter around 4.06 mm, AAT (≥5°C) of 1156.1°C and EAT (≥5°C) of 796.6°C before transplant were necessary for cultivar XiaHei to response the cold environment and begin the process of vernalisation, then bolting in the next year. Those results were almost the same with Gao’s [16] report that thresholds of pseudostem diameter and AAT (≥5°C) were 4 mm and 616°C for the Welsh onion’ bolting. For cultivar XiaHei, no bolting was observed when sown on 29 September, and transplanted in cold shed, bolting was observed when sown on 29 September, and transplanted in plastic tunnel, it was because that after transplanted, the temperature in the plastic still suitable for the Welsh onion’s growth, which made the Welsh onion reach the threshold of seedling age, AAT and EAT, then sense the low temperature and begin the vernalisation process, and bolting next year.

There are reports concerning the effects of cultivar and sowing date on the bolting of many crops, such as onion [26,27], hemp [28], lettuce [29] and lupin [30], among others. However, there are fewer reports concerning the effect on bolting of the transplant location, which is an important factor for bolting under practical production conditions. Moreover, limited research has been conducted on the factors that affect the bolting characteristics of the Welsh onion. Our research analysed the influence of the above three factors and their interactions on the bolting of Welsh onion and produced results consistent with the previous data. In our study, the bolting behaviour depended directly on the sowing date and the cultivar, with earlier sowing significantly increasing the bolting rate, and there was a considerable difference among the cultivars in their propensity to bolt. Ten major cultivars of the Welsh onion from North China were initially collected for this study, which included more cultivars than previous research and cultivars with multiple types of bolting characteristics. Finally, our results highlighted the importance of the transplant location for regulating the bolting behaviour. Use of the plastic tunnel as the transplant location for the Welsh onion during the winter produced heavier bolting in the following spring than occurred in the open field and the cold shed locations.

The mechanism responsible for the different bolting characteristics of the cultivars is still unknown. Few studies have been conducted on the inheritance of bolting resistance. Bolting resistance may be highly addictive because bolting-resistant cultivars have been developed by evolution. Several hypotheses may explain the mechanism of bolting resistance. First, bolting-resistant plants may require a larger critical plant size than bolting-susceptible plants to become receptive to a low-temperature stimulus. Second, bolting-resistant plants may require more chilling hours than are required by bolting-susceptible plants [26]. Our results demonstrated that the JinGuan (C_1_) and TieGanWuTong (C_7_) cultivars were susceptible to bolting but showed no tendency for small sizes compared with plants of the other cultivars, whereas the XiaHei (C_3_) and LinBang (C_10_) cultivars were resistant to bolting but were no larger than plants of the other cultivars. These patterns suggest that the second hypothesis is a more appropriate explanation for the different bolting behaviours of the ten Welsh onion cultivars, namely, that the bolting-resistant plants may require more chilling hours than the plants susceptible to bolting.

There are some reports about photoperiod’s effect on flowering of many crops [31-35]. Yamasaki [10] reported that Yakuwa and Koshimizu (1969) had found that the Welsh onion has a short-day requirement for flower-bud formation and Yamasaki and Miura (1995) found that the short-day was required only when the plant was not sufficiently exposed to low temperature. However, in Welsh onion’s practical production, the photoperiod of the natural environment is a constant value in a certain date of one year. Not like the factors, such as cultivar, sowing date, and transplant location which are mentioned above, could be artificially controlled. Thus, the photoperiod’s effect on bolting of Welsh onion was not discussed in this paper.

## Conclusion

This study revealed that the cultivar, sowing date, transplant location and their interactions influence the bolting of the Welsh onion. The conditions that resulted in late bolting and a low bolting rate were the use of a cultivar resistant to bolting, late sowing and transplantation in a low-temperature location. Thus, for the prevention of bolting in Welsh onion production in North China, the data suggested the choice of the XiaHei and LinBang cultivars, sowing at the end of September and transplantation in an open-field location. To enhance vernalisation for the seed industry, the data suggested that sowing be conducted in late August and that a plastic tunnel be used as the transplant location. At present, it is difficult for us to work out precisely the threshold values of seedling age and temperature for Welsh onion to through vernalisation because this experiment was carried out under natural field conditions. Therefore, future research, such as experiments with conditioned chambers to control the growth impact factors, needs to be done in the following days, in order to address the specific influence of transplant size and temperature on the bolting behaviour of the Welsh onion and establish models for predicting the bolting behaviour under various production conditions.

## Methods

### Site description and experimental design

The experimental field was located at the Horticultural Farm (34° 16' N, 108° 4' E) of the College of Horticulture, Northwest A&F University, Yangling, Shannxi Province, P. R. China. The trials were conducted in 2010–2011 and 2011–2012.

In 2010, ten cultivars (JinGuan, TieGanJuCong, XiaHei, LuCong, JiWang, YeFu, TieGanWuTong, JinGan, LvFeng and LingBang, which are abbreviated as C_1_ through C_10_, respectively) were sown on 3 dates (20 August, 9 September and 29 September, abbreviated as D_1_, D_2_ and D_3_, respectively) in the open field. One hundred healthy plants of each cultivar and sowing date were selected and transplanted on 19 November to three different locations (an open field, a cold shed and a plastic tunnel, abbreviated as L_1_, L_2_ and L_3_, respectively). The plastic tunnel was 50 m long, 8 m wide and 3.5 m high, and the cold shed was a kind of small plastic tunnel, 5 m long, 3 m wide and 2.4 m high. Both cold shed and plastic tunnel were treated as keeping warm facilities in north China without heating device. The light density and the photoperiod of cold shed and plastic tunnel were almost the same compared to the open field.

Each transplant plot was 4 m long and 10 cm wide. The plants were transplanted into a space of 4 cm × 5 cm at a depth of 3–4 cm after each plot was tilled and fertilised with cattle manure at 5 kg/m^2^. Both N and P were each applied at 60 g/plot as ammonium hydrogen carbonate and calcium superphosphate, respectively. Hand weeding was done during plant growth. The plants were irrigated immediately after transplantation and subsequently, as needed.

The temperature of seedling period and in the three transplant locations were recorded hourly using a computerised data logger (TM-01, YinMeng Electronic Co. Ltd., Handan, Henan, China) during the course of the experiment. Before transplantation, the plant height, pseudostem diameter and number of leaves were measured after randomly sampling 20 plants per plot. A visible scape (length >5 mm) was regarded as an indicator of bolting initiation. The bolting by individual plants was documented by visual inspection of each plot every 3 days. At the end of the experiment, the number of plants that apparently had not bolted by 30 April, 2011, was recorded, and the percentage of plants that bolted within each treatment was calculated.

The experiment was repeated the next year, i.e., 2011–2012, but only the cultivars C_1_, C_3_, C_6_, C_7_, C_8_ and C_10_ were selected for planting based on the previous results.

### Statistical analyses

The experiment was conducted in a randomised complete block design with three replicates. The data were assessed by analysis of variance (ANOVA) using the SAS software (SAS Institute Inc., Cary, NC), and the least significant difference (LSD) test at P < 0.05 was used for multiple comparisons among the treatment means.

AAT = Sum of Td when Td ≥ 5°C, 10°C or 15°C

Td: Daily Mean Temperature

Unit: deg.

EAT = AAT - Lt * C

Lt: 5°C, 10°C or 15°C; C: days of Td above 5°C, 10°C or 15°C

Unit: deg.

EAT = Sum of Td when Td ≤ 13°C

Td: Daily Mean Temperature

Unit: deg.

EALT = Ht * C - EAT

Ht: 13°C; C: days of Td under 13°C

Unit: deg.

## Endnotes

^a^ Different letters indicate significant differences between means at the 5% probability level by LSD (Table 6).

### Availability of supporting data

The onion’s planted area and production data in 2011 form FAOSTAT was attached as availability of supporting data.

## Abbreviations

AAT: Active accumulated temperature; EAT: Effective accumulated temperature; ALT: Accumulated low temperature; EALT: Effective accumulated low temperature; C1: JinGuan; C2: TieGanJuCong; C3: XiaHei; C4: LuCong; C5: JiWang; C6: YeFu; C7: TieGanWuTong; C8: JinGan; C9: LvFeng; C10: LingBang; D1: 20 August; D2: 9 September; D3: 29 September; L1: Open field; L2: Cold shed; L3: Plastic tunnel.

## Competing interests

The authors declare that they have no competing interests.

## Authors’ contributions

YD carried out the mainly research work of the manuscript, including experiment design, plant cultivation, and statistical analysis. ZC proposed the research proceeding and statistical analysis method and modified this manuscript until submitted. HM participated in the plant cultivation and statistical analysis process. HL participated in the design of the study, did the mainly physical work, and performed the statistical analysis. CW conceived of the study, and participated in its design and coordination and helped to plant cultivation and statistical analysis. ARK provided statistical analysis and writing assistance help. All authors read and approved the final manuscript.

## Authors’ information

Zhihui Cheng and Huanwen Meng are supervisors of other authors, working at Northwest A&F University; Yinxin Dong, Cuinan Wu, and Abdul Rehman Khan are Ph.D students of Northwest A&F University, studying on plant of the genus Allium, such as the Welsh onion, onion, garlic, et al.; Hanqiang Liu is a postgraduate student of Northwest A&F University, studying on plant of the genus Allium too.

## Supplementary Material

Additional file 1 Onion harvested area and production in 2011-FAOSTAT. Click here for file
